# Enhancing electrospray ionization efficiency for particle transmission through an aerodynamic lens stack

**DOI:** 10.1107/S1600577524000158

**Published:** 2024-02-02

**Authors:** Safi Rafie-Zinedine, Tej Varma Yenupuri, Lena Worbs, Filipe R. N. C. Maia, Michael Heymann, Joachim Schulz, Johan Bielecki

**Affiliations:** a European XFEL, Holzkoppel 4, 22869 Schenefeld, Germany; b Institute of Biomaterials and Biomolecular Systems, University of Stuttgart, Pfaffenwaldring 57, 70569 Stuttgart, Germany; c Laboratory of Molecular Biophysics, Department of Cell and Molecular Biology, Uppsala University, Husargatan 3 (Box 596), 75124 Uppsala, Sweden; Paul Scherrer Institut, Switzerland

**Keywords:** electrospray ionization, aerosol injection, aerosol neutralization, single-particle imaging, XFELs

## Abstract

This work investigates the performance of the electrospray aerosol generator at the European X-ray Free Electron Laser (EuXFEL). This generator is, together with an aerodynamic lens stack that transports the particles into the X-ray interaction vacuum chamber, the method of choice to deliver particles for single-particle coherent diffractive imaging (SPI) experiments at the EuXFEL. For these experiments to be successful, it is necessary to achieve high transmission of particles from solution into the vacuum interaction region. Particle transmission is highly dependent on efficient neutralization of the charged aerosol generated by the electrospray mechanism as well as the geometry in the vicinity of the Taylor cone. We report absolute particle transmission values for different neutralizers and geometries while keeping the conditions suitable for SPI experiments. Our findings reveal that a vacuum ultraviolet ionizer demonstrates a transmission efficiency approximately seven times greater than the soft X-ray ionizer used previously. Combined with an optimized orifice size on the counter electrode, we achieve >40% particle transmission from solution into the X-ray interaction region. These findings offer valuable insights for optimizing electrospray aerosol generator configurations and data rates for SPI experiments.

## Abstract 2

We investigate the performance of an electrospray aerosol generator at the European X-ray Free Electron Laser for single-particle coherent diffractive imaging. The findings highlight enhanced particle transmission efficiency with VUV ionization, larger orifice diameters, electric fields and 3D-printed twin-nozzle design, providing insights for optimizing aerosol injection in XFEL experiments.

## 1. Introduction

Methods for delivering a dense beam of single particles to the interaction region of a free-electron laser (FEL) are of significant interest in uncovering the structural properties of small nanometre-scale particles. Commonly employed methods for achieving this include liquid jet injection and gas phase injection. Among these methods, gas phase injection through an aerodynamic lens stack (ALS) offers several advantages. It provides increased scattering contrast, reduced background scattering and the ability to collect data at high rates. In particular, aerosolized particle delivery provides an opportunity to harness the unique capabilities of the European XFEL (EuXFEL), which emits X-ray ‘trains’ at 10 Hz, each containing up to 2700 pulses separated by 220 ns (Decking *et al.*, 2020 ▸). The high repetition rate allows for rapid acquisition of large datasets, provided that the sample-delivery system is compatible with the XFEL pulse structure. Aerosol injection can meet the latter requirement, where gas-phase particles can achieve velocities ranging from tens to hundreds of metres per second, thereby preventing multiple exposures from subsequent pulses (Hantke *et al.*, 2018 ▸; Bielecki *et al.*, 2019 ▸).

There are several aerosolization methods available for gas phase injection through an ALS, including electrospray (ES), atomizers, gas dynamic virtual nozzles (GDVNs) and others. However, ES stands out among these methods due to its capability to produce smaller droplets, which results in virtually contaminant-free sample delivery (Bielecki *et al.*, 2019 ▸). ES is a powerful technique that uses electrical forces to atomize liquids, transforming them into nanometre to micrometre-sized droplets. This process is accomplished by overcoming the liquid surface tension with electric forces as the liquid flows through a capillary nozzle. There are several forms of ES, including ‘grinded’ single-capillary, dual-capillary and tri-nozzle ES, with single-capillary ES being the most extensively researched and documented (Zeleny, 1914 ▸; Chen *et al.*, 1995 ▸; de la Mora, 2007 ▸).

ES systems typically consist of a spray nozzle positioned opposite a plate, with or without an orifice at the center. By applying an electric field between the nozzle and the plate, a cone-jet meniscus, called a Taylor cone, forms when the liquid surface tension is counterbalanced by the electric force. The liquid jet undergoes Rayleigh breakup into a mono-disperse droplet stream, which has gained significant interest for its potential applications (Jaworek & Krupa, 1999 ▸) such as drug delivery (Enayati *et al.*, 2010 ▸), nanoparticle material synthesis (Jaworek, 2007 ▸), thin-film deposition, particle encapsulation (Bock *et al.*, 2012 ▸), surface coating, agricultural treatments, fuel spraying, ink-jet printers and colloid micro-thrusters (Jaworek & Sobczyk, 2008 ▸).

**Figure 1:**
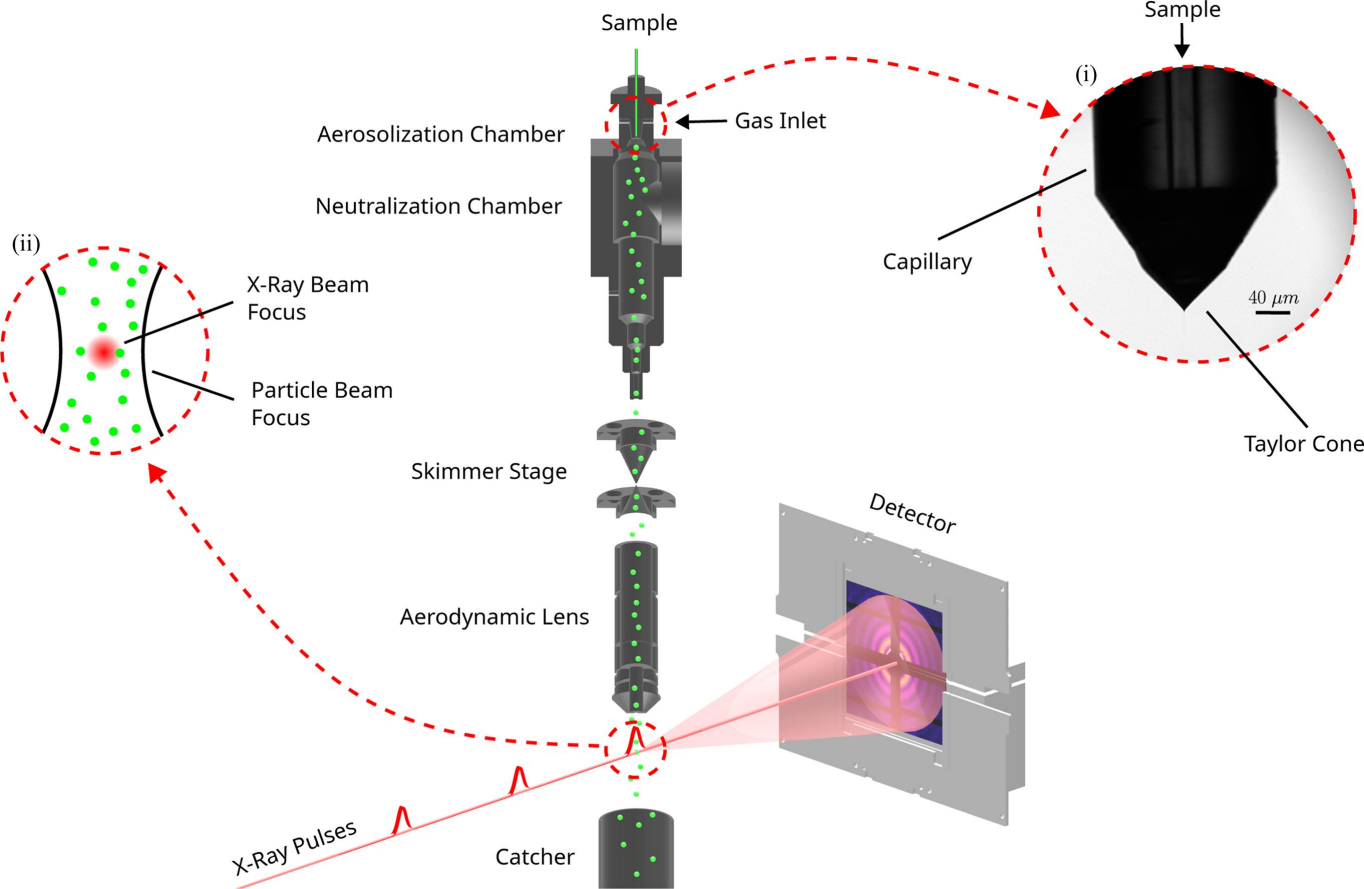
Schematic of the experimental setup of an ES-based aerosol injector used at the EuXFEL. The injector includes the ES process depicted at the top, where the sample is aerosolized. The aerosol beam then passes through the skimmer stages and the aerodynamic lens before reaching the interaction chamber, where it is hit by the XFEL beam. The XFEL pulses are scattered off particles in the aerosol beam to produce diffraction patterns on the detector. Insets: (i) a typical Taylor cone, here injecting silver nano-cube in 80 m*M* ammonium acetate dissolved in ethanol; (ii) the X-ray beam focus is significantly smaller than particle beam focus.

Our primary focus in this study is the application of ES as a particle-delivery system for single-particle imaging (SPI) experiments at free-electron X-ray laser facilities (Bielecki *et al.*, 2019 ▸; Bogan *et al.*, 2008 ▸). In these experiments, shown schematically in Fig. 1 ▸, the droplet stream from the ES is allowed to evaporate in order to transfer the sample particles from solution into the gas phase. Subsequently, the particles pass through an ALS (de la Mora & Riesco-Chueca, 1988 ▸; Liu *et al.*, 1995 ▸), described in detail by Hantke *et al.* (2018 ▸), which creates a focused particle beam that intersects the pulsed X-ray beam. Each X-ray pulse contains enough photons to produce a diffraction pattern of a single particle, while the pulse duration of ∼10 fs has been considered short enough to enable ‘diffraction before destruction’ (Neutze *et al.*, 2000 ▸). The information in each pattern can be used to obtain 2D, or in some cases pseudo-3D, information about individual particles in an ensemble. If the particles are reproducible, it is also possible to merge the individual patterns into a 3D volume in reciprocal space. After phase retrieval, one can obtain the full 3D structural information about the particle. Numerous such experiments have been performed at FELs on cells (van der Schot *et al.*, 2015 ▸), viruses (Seibert *et al.*, 2011 ▸; Ekeberg *et al.*, 2015 ▸; Kurta *et al.*, 2017 ▸; Rose *et al.*, 2018 ▸; Munke *et al.*, 2016 ▸; Reddy *et al.*, 2017 ▸; Daurer *et al.*, 2019 ▸; Sobolev *et al.*, 2020 ▸; Gorkhover *et al.*, 2018 ▸; Lundholm *et al.*, 2018 ▸), cell organelles (Hantke *et al.*, 2014 ▸), proteins (Ekeberg *et al.*, 2022 ▸) and inorganic nano-particles (Ho *et al.*, 2020 ▸; Ayyer *et al.*, 2021 ▸). Despite many successful imaging experiments, concerns persist regarding particle transmission efficiency, especially as the sample size decreases (Bielecki *et al.*, 2020 ▸).

Although substantial research has explored droplet aerosolization in ES processes (Taylor, 1964 ▸; Wilm & Mann, 1994 ▸; Gañán-Calvo, 1997 ▸), further study is necessary to optimize particle neutralization and transmission at various stages. Initially, the spray is narrow, but the Coulomb repulsion of highly charged droplets causes the spray to widen and increases the likelihood of deposition on the counter electrode. To transport ES-generated particles through a carrier gas flow, it is essential to neutralize the charged particles. This can be accomplished by exposing the particles to an environment with ions produced using methods such as radioactive sources, corona discharge and vacuum ultraviolet (VUV)/soft X-ray irradiation (Ebeling *et al.*, 2000 ▸; Fu *et al.*, 2011 ▸; Chen *et al.*, 1995 ▸; Liu & Chen, 2014 ▸). Each of these techniques presents its own challenges and limitations, such as strict regulations for radioactive materials, high-voltage power supplies, potential ozone production for corona discharge and complicated safety regulations for operating soft X-ray ionizers. VUV radiation sources, in contrast, offer an accessible, safe and efficient alternative for bipolar ion generation. In comparison with other methods, VUV sources are easily purchased, operated and integrated into various applications. Their compact size and portability make them an ideal candidate for particle charge reduction.

In a previous study on ES transmission efficiency by Fu *et al.* (2011 ▸), it was highlighted that the highly charged nature of particles produced by ES leads to significant particle loss at the counter electrode orifice plate due to Coulombic repulsion between the highly charged particles (Fu *et al.*, 2011 ▸). It was found that ES transmission could be improved by increasing either the bi-polar charge density from the neutralizer, the carrier gas flow and/or the orifice diameter in the counter electrode. Thus, we primarily optimized the performance of our sample-delivery system by varying the types of ionizers (VUV, soft X-ray and ^210^Po) as well as the geometry of the ES setup. However, the limitations on gas flow rate for single-particle imaging made it difficult to explore higher rates as in prior studies.

Utilizing advancements in two-photon polymerization, a cutting-edge technique for creating intricate 3D fluidic devices with submicrometre accuracy, can significantly improve microfluidic nozzle design. This innovative approach has the potential to transform the development and production of microfluidic nozzles, resulting in enhanced performance, miniaturization and customization possibilities (Knoška *et al.*, 2020 ▸; Vakili *et al.*, 2022 ▸). By employing this technique, tailored microfluidic nozzles can be designed to generate two Taylor cones simultaneously. Using this technique, we designed a customized ‘twin nozzle’ capable of producing dual Taylor cones. We tested it against a grinded single nozzle to enhance particle production and delivery. The results have implications for XFEL single-particle imaging and future injector development.

## 2. Experimental setup and methods

### 2.1. Configurations of electrospray aerosol generator

**Figure 2:**
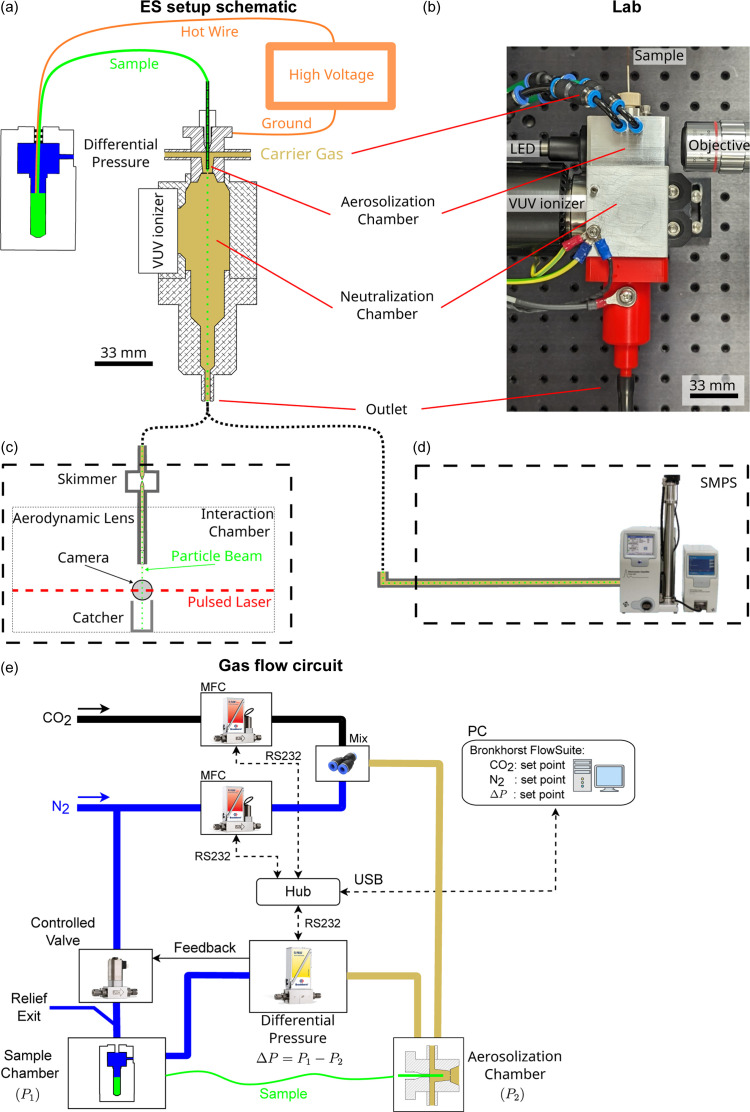
Schematic of the experimental setup used to measure particle transmission efficiency. (*a*) Schematic of the ES setup, in which the sample is aerosolized by forming a Taylor cone. The aerosol is then carried to the neutralization chamber, where it is neutralized by a VUV ionizer before exiting either through a Rayleigh scattering setup or an SMPS setup. (*b*) Layout of the ES setup as arranged in the laboratory. (*c*) Rayleigh scattering setup, which comprises double skimmer stages that remove excess gas and an aerodynamic lens that focuses the particle beam before it passes through the interaction chamber and is detected by a pulsed laser beam. (*d*) SMPS setup, which uses a series of differential mobility analyzers and a condensation particle counter to analyze the sample. (*e*) Schematic of the gas flow circuit, which incorporates gas flow meters, differential pressure and valves to regulate the flow rate of the sample and carrier gases.

Fig. 2 ▸ illustrates the design of an ES aerosol generator incorporating a VUV ionizer with emission sharply peaked at λ = 155 nm. The generator is composed of two primary chambers: the aerosolization and neutralization chambers, which are divided by an orifice disk plate. A grinded single glass capillary featuring an inner diameter (ID) of 40 µm and an outer diameter (OD) of 360 µm serves as the spray nozzle in the aerosolization chamber. The sample reservoir is biased at a positive high voltage, while the orifice plate, aerosolization chamber and neutralization chamber are electrically grounded. The distance between the capillary tip and the grounded orifice plate is kept constant at around 1 mm. N_2_ mixed with CO_2_ to prevent corona discharge formation was used as the particle carrier gas. Mass flow controllers (Bronkhorst model F-201CV) independently control the two gas flow rates. A differential pressure (Δ*P*) meter (Bronkhorst model P-506C) coupled with direct-operated control valves (Bronkhorst model F-001) regulates Δ*P* across the capillary to control the liquid flow rate. An optical system, including a fiber-coupled LED light source, infinity-corrected objectives (5X Mitutoyo Plan Apo *f* = 200), a tube lens (Thorlabs TTL200-A) and a CCD camera (Basler acA2440-20gm) enables high-resolution observation of the Taylor cone in the aerosolization chamber (Fig. 1 ▸). The neutralization chamber includes a cylindrical interaction region measuring 25.2 mm in diameter, which facilitates the interaction between ionizer radiation and the particle beam along with its carrier gas.

Five distinct configurations of the ES aerosol generator were tested and compared in this study, including four configurations for optimizing particle transmission efficiency and one for enhancing particle production. The emitted droplet sizes were characterized previously (Vakili *et al.*, 2022 ▸). The baseline configuration is a single ES, 0.5 mm counter electrode orifice, an N_2_ volumetric flow rate of 1 l min^−1^, a CO_2_ volumetric flow rate of 0.3 l min^−1^ and a deuterium-based VUV lamp as the neutralizer (Hamamatsu L12542) positioned at a 90° angle relative to the particle beam [Fig. 4(*a*)]. The emission spans wavelengths from 115 nm to 400 nm. The second configuration [Fig. 4(*b*)] replaces the neutralizer with a soft X-ray source (Hamamatsu L12645) whose emission peaks at around 5 keV. An additional spacer of 36 mm between the orifice plate and neutralization chamber is included in this configuration to prevent X-ray photons from discharging the Taylor cone directly. The third configuration mirrors the baseline configuration, but with a negatively biased electrode at the end of the neutralization chamber intended to pull positively charged droplets away from the orifice disk [Fig. 5(*a*)]. The fourth configuration has a larger 1 mm orifice diameter [Fig. 6(*a*)]. Finally, the fifth configuration operates with a 3D-printed twin nozzle capable of producing two Taylor cones simultaneously [Fig. 7(*b*)].

In the data collection process we alternated between different ES configurations to ensure reproducibility of our results and we ensured that the Taylor cone remained stable and consistent across all configurations. The gas flow rate was set to the maximum level appropriate for SPI experiments, taking into account gas mass flow constraints for safe X-ray detector operation. Lower gas flow rates resulted in reduced transmission efficiency and were not investigated further.

The ES was operated using a capillary with a fixed ID *D* = 40 µm and length *L* = 30 cm. A stable cone-jet mode was maintained for all tests by adjusting the voltage bias relative to the counter electrode. The CO_2_ flow rate was fixed at 0.3 l min^−1^. The sample flow rate *Q* was controlled between 433 nl min^−1^ and 866 nl min^−1^, respectively, by varying Δ*P* according to the Hagen–Poiseuille equation,

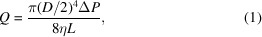

where η is the viscosity of the fluid.

The twin nozzle was designed using the Siemens NX software, featuring two capillary inlets compatible with 360 µm OD fused silica capillaries and dual outlets (Fig. 7). The design was exported in STL format. Print-job instructions, or GWL files, were generated using the Nanoscribe *DeScribe* software (Nanoscribe GmbH, Germany). To enhance structural stability, we used a solid-volume printing strategy with 1 µm slicing and 0.5 µm hatching. Printing was conducted on the Nanoscribe Photonic Professional GT, using IP-S photoresist. Post-printing, the device was immersed in propylene glycol methyl ether acetate (PGMEA) for 1–2 days to remove uncured material. After this development phase, the devices were rinsed in 2-propanol twice for 30 min each time and then air-dried on a cleanroom cloth. Assembly was done on a polydimethylsiloxane (PDMS) sheet and observed under an optical microscope. Two 360 µm OD fused silica capillaries were inserted in the inlets and secured with 5 minute epoxy (from Devcon). These were routed through hollow 1/16 inch OD stainless-steel tubing (IDEX U-145, ID 0.046 inches) and affixed in place. The 3D model of the twin nozzle is publicly accessible and can be downloaded from the GitHub repository: https://github.com/safirafie/ESDesign.

### 2.2. Characterization and sample-delivery setup

**Figure 3:**
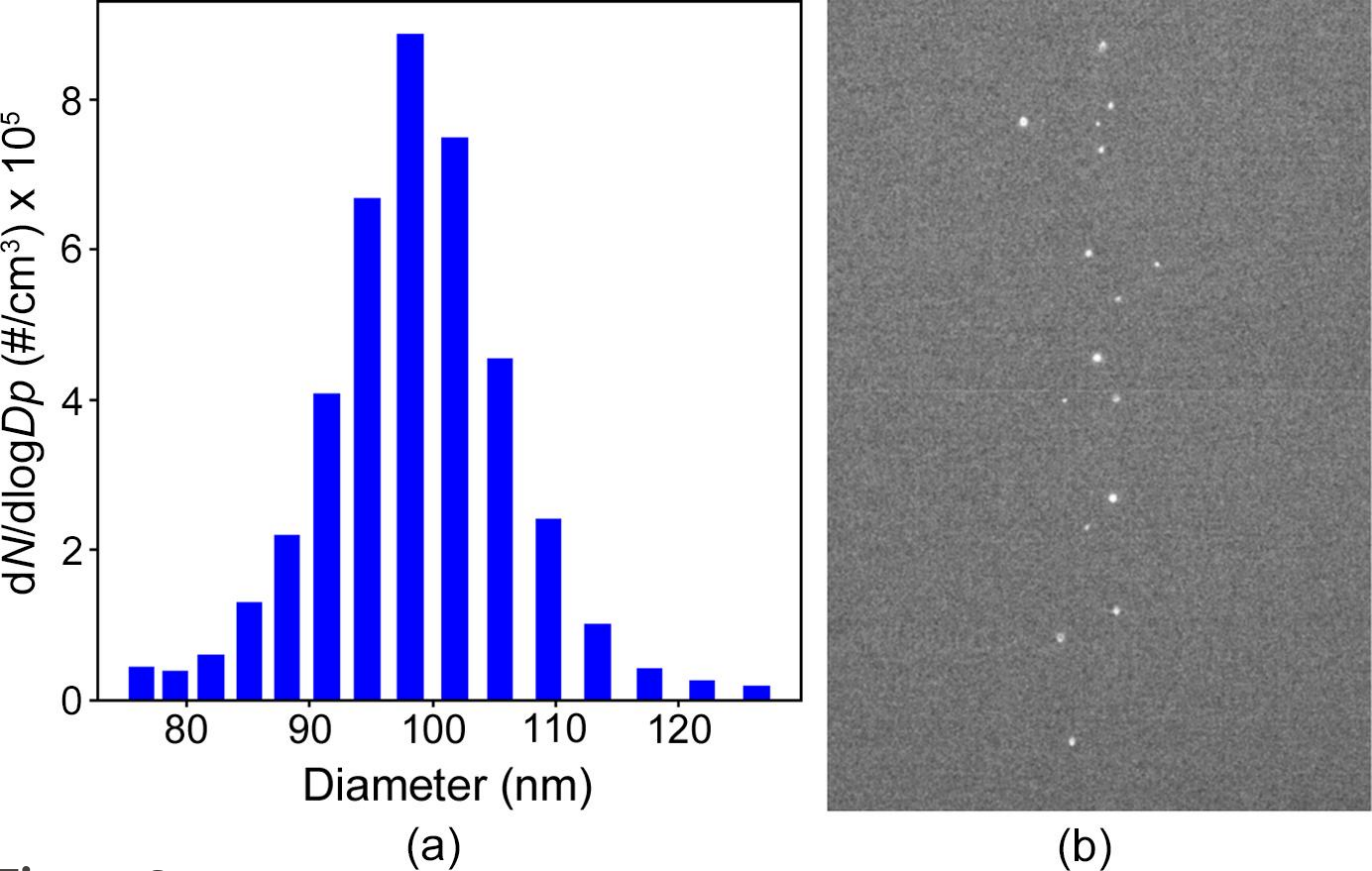
Characterization methods using the baseline configuration with a silver nano-cube in ethanol buffer. (*a*) Particle distribution from SMPS. (*b*) Frame from Rayleigh scattering setup.

Characterization of the ES aerosol generator was conducted using a scanning mobility particle sizer (SMPS) setup (TSI 3082 Electrostatic classifier, TSI 3788N-WCPC, TSI, Shoreview, MN, USA) and a Rayleigh-scattering setup (Hantke *et al.*, 2018 ▸) (Fig. 2 ▸). For each measurement, the generation system outlet was initially connected to the SMPS setup to record size distribution spectra [Fig. 3 ▸(*a*)]. Subsequently, it was connected to the Rayleigh-scattering setup to image particles reaching the interaction chamber [Fig. 3 ▸(*b*)]. Then, the particle transmission efficiency was calculated for each configuration.

In addition to particle losses from the ES generator, particles can potentially be lost in the two skimmer stages as well as at the orifices inside the aerodynamic lens. To account for these losses, we performed Rayleigh scattering measurements to measure the absolute particle transmission through the whole sample-delivery system. The particles exit the aerodynamic lens into a vacuum chamber at approximately 1 × 10^−4^ mbar where they intersect a λ = 532 nm double-pulsed Nd:YAG laser producing 6.5 ns-long pulses each with 100 mJ pulse energy. A plano-convex lens with a 150 mm focal length focused the laser beam to a 100 µm full width at half-maximum (FWHM) spot at the intersection point with the particle beam. Scattering from aerosol particles was imaged with a microscope situated outside the vacuum chamber, comprising an infinity-corrected objective lens, a zoom lens and a scientific CMOS camera (Zyla 4.2 sCMOS).

Particle velocities (*v* = *L*/τ) were measured by illuminating each particle twice, with time separation τ, resulting in two particle detections separated by a distance *L* within the same camera exposure. Images obtained from the Rayleigh scattering were analyzed using an open-source Python code (https://github.com/safirafie/SPCounting) which calculates *v* and the position *x*, *y* for each detected particle.

The particle transmission *T* was calculated as the ratio between the flux of particles exiting the aerodynamic lens to the theoretical flux generated by the ES, 

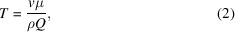

where μ is the linear density of particles detected with Rayleigh scattering and ρ is the particle concentration in solution (Hantke *et al.*, 2018 ▸).

The sample utilized in this study was a commercial silver nano-cube with a side length of 75 nm. The particles were suspended in an ethanol-based 80 m*M* ammonium acetate buffer at the concentration ρ = 1.9 × 10^10^ ml^−1^.

## 3. Results and discussion

### 3.1. Effects of different ionizers

**Figure 4:**
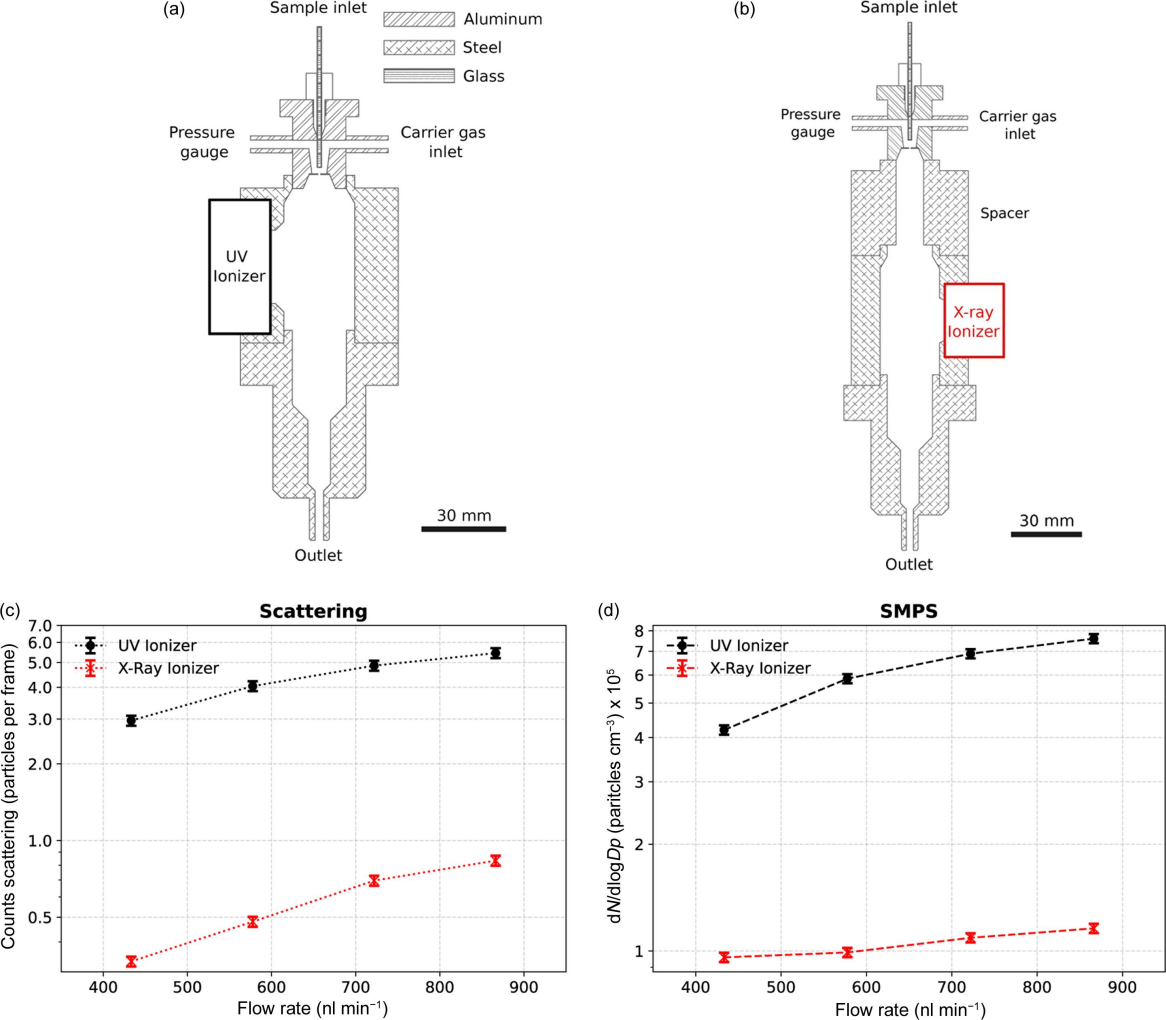
Comparison of the particle transmission efficiency in ES using X-ray and VUV ionizers for different sample flow rates. Schematic setup of the (*a*) VUV and (*b*) X-ray ionizers. Particle counts in each of these setups measured using (*c*) Rayleigh scattering and (*d*) SMPS, respectively. The results show that the VUV ionizer has higher particle transmission compared with the X-ray ionizer.

We investigated the impact of two distinct ionization techniques on particle transmission efficiency in the ES process: the VUV ionizer (baseline configuration), as illustrated in Fig. 4 ▸(*a*); and the X-ray ionizer, as depicted in Fig. 4 ▸(*b*). In the absence of an ionizer, the ES process generates positively charged particles that are influenced by the electric field. Consequently, these particles tend to accumulate on grounded surfaces, such as the faces of the orifice plate and the internal surface of the neutralization chamber. The accumulation of charged particles on these surfaces can lead to nearly complete losses in particle transmission, severely reducing the efficiency of the ES system.

Everything else being equal, the VUV ionizer configuration delivers approximately seven times more particles than using the X-ray ionizer, as measured by both Rayleigh scattering and SMPS [Figs. 4 ▸(*c*) and 4 ▸(*d*)]. Considering the calculated particle velocity 28 m s^−1^, we obtain 35% and 5% transmission efficiency with the VUV and X-ray setups, respectively.

We mainly attribute the increased particle transmission to the fact that VUV photons are completely absorbed by the carrier gas in the direct vicinity of the emission window. In contrast, a majority of the X-ray photons do not interact with the carrier gas inside the neutralization chamber (Seltzer, 1995 ▸). This leads to a higher number of charges produced, and hence more efficient neutralization, with the VUV source. For the same reason, the VUV source can be placed closer to the Taylor cone without disturbing its operation, leading to a larger concentration of neutralizing charges close to the counter electrode orifice plate.

Another commonly used ionizer in ES applications is the radioactive ^210^Po α particle source. We installed a commercial ^210^Po ionizer in a similar configuration to the baseline configuration. We found that the ^210^Po ionizer outperformed the X-ray ionizer by approximately a factor of 3. However, it underperformed when compared with the VUV ionizer by approximately a factor of 2.4.

### 3.2. Effects of using an auxiliary electric field

**Figure 5:**
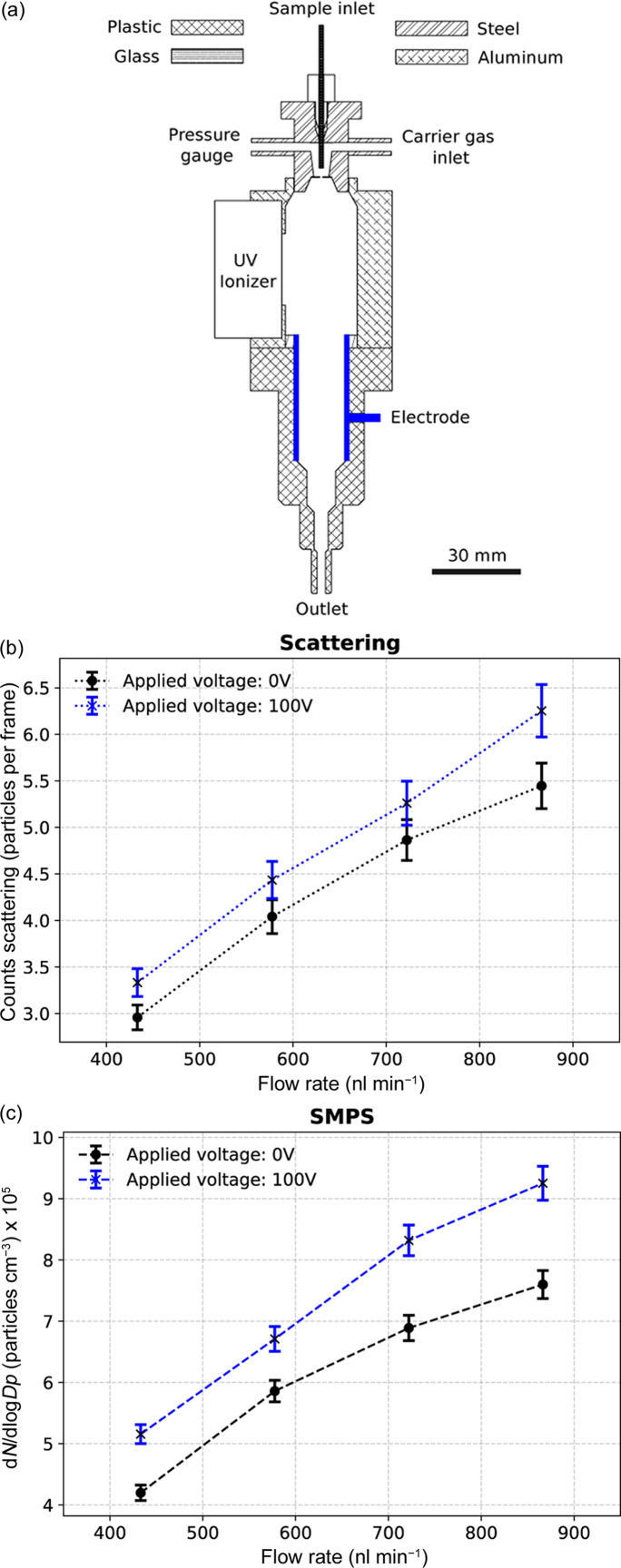
ES transmission efficiency using a VUV ionizer with an auxiliary electrode operating at 0 V or −100 V. (*a*) Schematic of the setup with the opposing electrode highlighted in blue. Particle counts were measured with (*b*) the Rayleigh scattering setup or (*c*) SMPS. Applying −100 V on the opposite electrode increased particle transmission marginally.

We hypothesized that the presence of the auxiliary electric field can assist in attracting positively charged particles away from the counter electrode (Fig. 5 ▸). Applying −100 V to the electrode resulted in an approximate increase of 11% in particle transmission. At −50 V, −200 V, −500 V and −1000 V the transmission efficiency declined. For the circular electrode used in this study, there is an optimal electric field strength, which may arise from insufficient residence times in the ionization chamber or from radial components of the electric field.

### 3.3. Effects of orifice diameter

**Figure 6:**
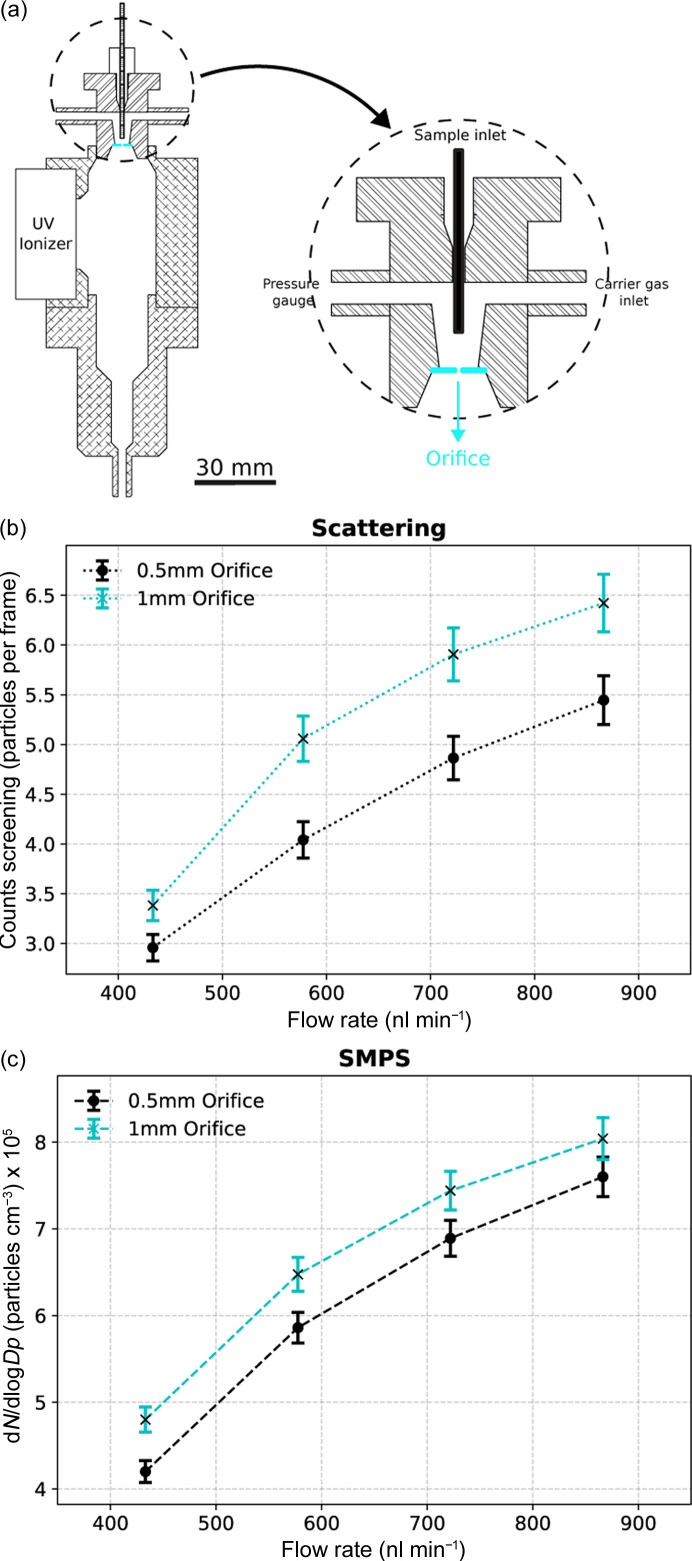
Particle transmission efficiency in ES using a VUV ionizer with 0.5 mm- and 1 mm-diameter orifices. (*a*) Schematic of the setup of the VUV ionizer, with the orifice highlighted in cyan. Particle counts in each of these setups were measured using (*b*) Rayleigh scattering and (*c*) SMPS.

Particle transmission with orifice plates of 0.5 mm, 1 mm and 2 mm in diameter were compared [Fig. 6 ▸(*a*)]. A 1 mm orifice plate gave approximately 17% more particles compared wth the 0.5 mm baseline configuration [Fig. 6 ▸(*b*)], which translates to 41% transmission efficiency. This is in agreement with the previous study by Fu *et al.* (2011 ▸), which also showed an improvement in transmission efficiency with the use of larger orifice sizes. However, in contrast a 2 mm orifice plate could not support a stable Taylor cone with our geometry and gas flow rates suitable for SPI experiments.

### 3.4. Effects of a multi-Taylor cone

**Figure 7:**
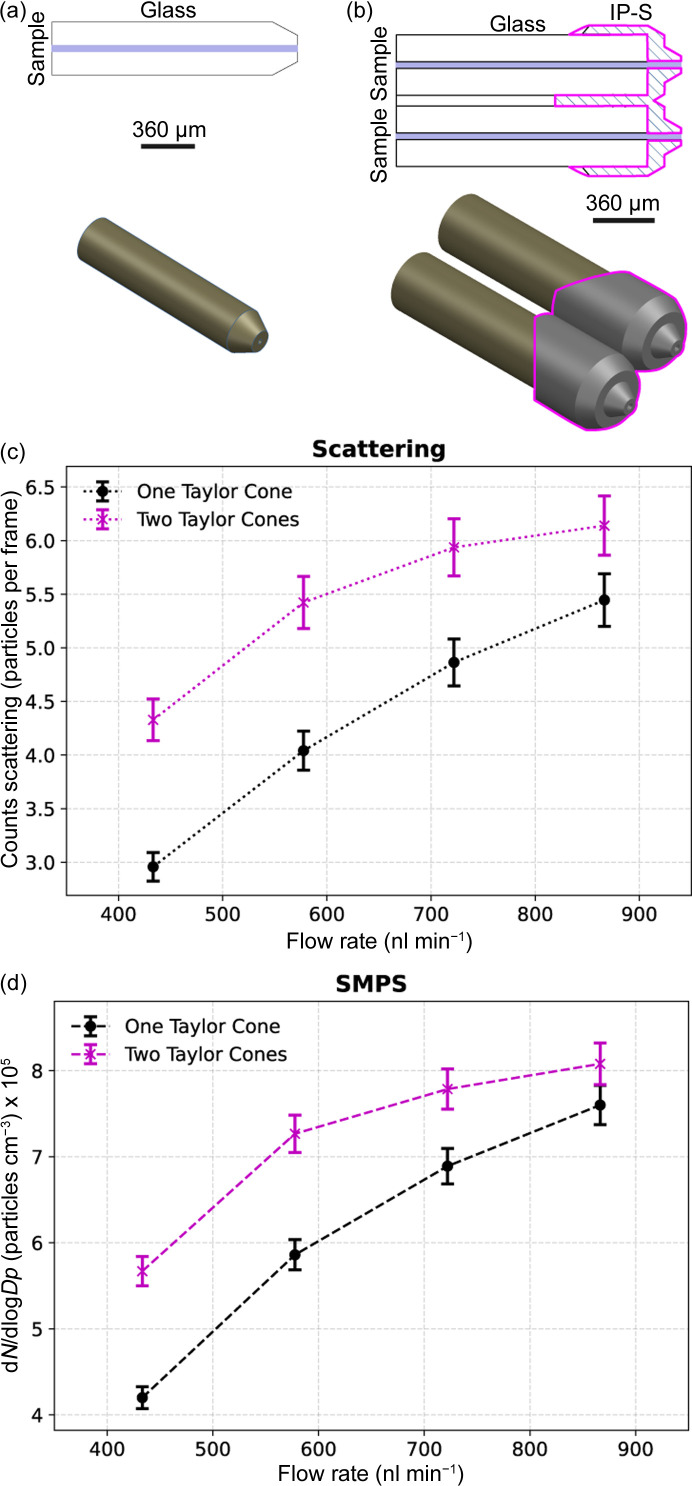
Particle transmission efficiency of ES using a VUV ionizer with different nozzle geometries: a grinded capillary that produces a single Taylor cone and a 3D-printed ‘twin nozzle’ featuring two separate openings, each generating its own Taylor cone. (*a*) Schematic of the ground nozzle. (*b*) Schematic of the twin nozzle with dual openings. (*c*) Particle counts using the Rayleigh scattering setup. (*d*) Particle counts using SMPS.

We designed a 3D-printed nozzle ‘twin nozzle’ to generate dual Taylor cones in an attempt to increase the total number of transmitted particles compared with the single-capillary emitter (Fig. 7 ▸). Particle delivery was approximately 28% higher in comparison with the case where the grinded nozzle was used. Although the multi-Taylor cone configuration increased the number of particles delivered into the inter­action chamber, the overall transmission efficiency was measured at 22%, which is significantly lower than the baseline efficiency. Optical imaging showed that the two cones were not aligned with the orifice in the counter plate, resulting in a non-optimal angle and an extended travel distance for the particles from the cones to the orifice exit.

### 3.5. Comparison of particle transmission efficiency

**Figure 8:**
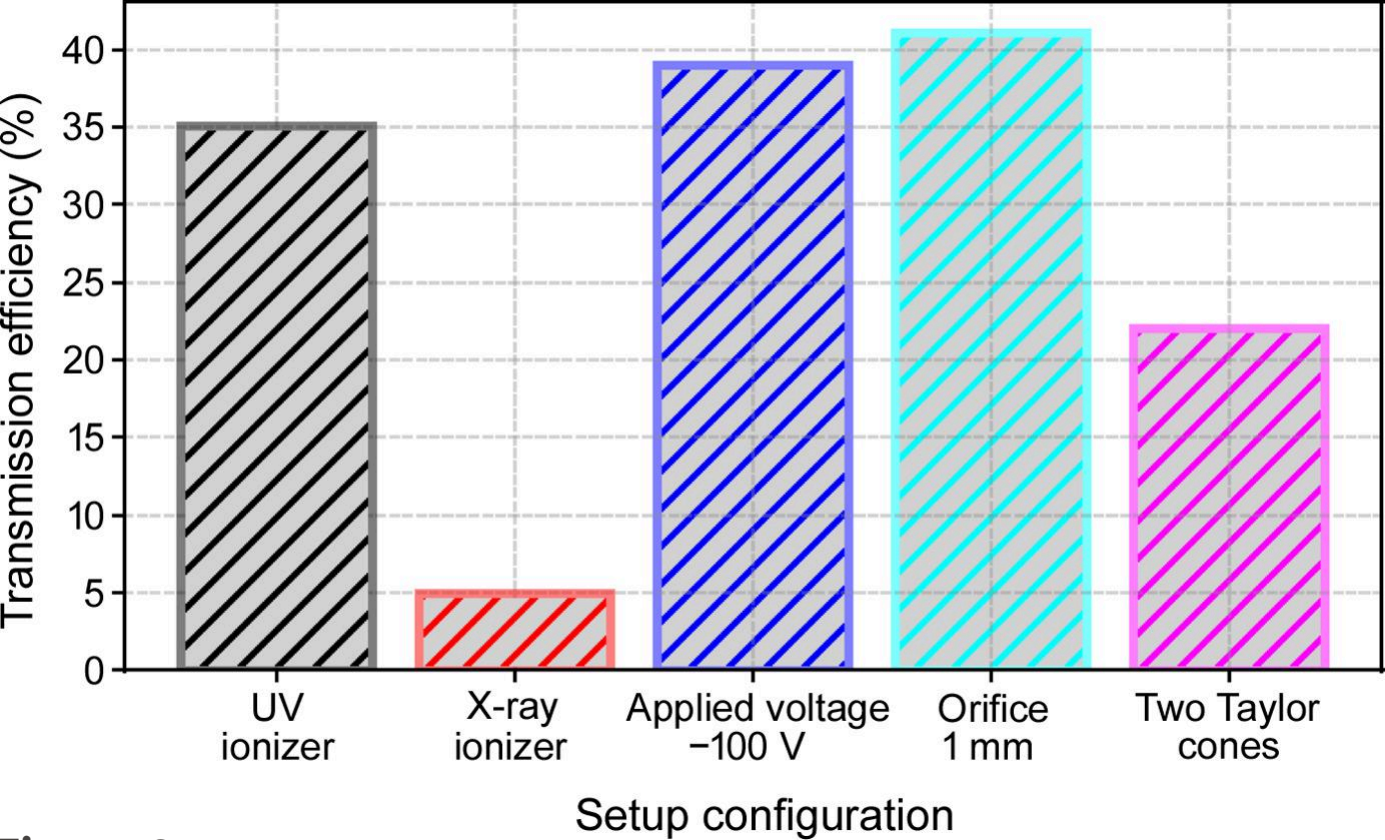
Comparison of particle transmission efficiency for various ES configurations, demonstratng the influence of different factors such as ionizer type, orifice plate size, the presence of an additional auxiliary electric field and the production of dual Taylor cones.

A comparison of the transmission efficiencies – calculated as the flow rate average – for all investigated configurations is shown in Fig. 8 ▸. No significant correlation between transmission efficiency and sample flow rate was found. Note that our ES aerosol generator is not the only source of particle losses in the aerosol sample-delivery system. Particle losses can also occur in the two-nozzle/skimmer stages and on the orifices used as aerodynamic lenses. This is especially true for lighter particles such as proteins or viruses, which closely follow the gas carrier streamlines. For such particles, Rayleigh scattering measurements require a tightly focused laser beam. On the other hand, the aerodynamic focusing is decreased, leading to a particle beam much larger than the required laser beam size. Thus, total transmission measurements become considerably more challenging with decreasing sample size.

## 4. Conclusions

To improve single-particle imaging experiments at FELs, five distinct aerosol sample-delivery configurations were systematically compared for their transmission efficiency. Rayleigh scattering and SMPS results were consistent in quantifying the effect of each configuration. Most importantly, VUV ionization increased the particle transmission by a factor of seven relative to X-ray ionization. An auxiliary electric field, or a larger orifice diameter of 1 mm each had a relatively minor positive effect on particle transmission. Employing a 3D-printed twin-nozzle to generate two Taylor cones considerably improved the particle number, while total transmission decreased. This highlights the potential of exploring innovative nozzle designs to improve the overall performance.

Further studies can focus on investigating additional parameters that may influence the performance of ES particle-generation systems. These may include electric field strength and distribution, different electrode configurations, and aerosolization chamber designs. Improved neutralization may be achieved with additional VUV ionizers, or refined ionizer geometry, in the neutralization chamber. Though we achieved particle transmissions only a factor of 2.5 away from an ideal 100%, measuring and optimizing transmission for smaller (<20 nm) and lighter particles remains an unmet need for single-particle imaging at FELs.
